# Supplementary figures and images for: Anti-BDCA2 monoclonal antibody inhibits plasmacytoid dendritic cell activation through Fc-dependent and Fc-independent mechanisms
Source: EMBO Mol Med. 2015 Mar 11;7(4):464–76. doi: 10.15252/emmm.201404719 (PMC4403047; doi:10.15252/emmm.201404719)

**Figure S1**

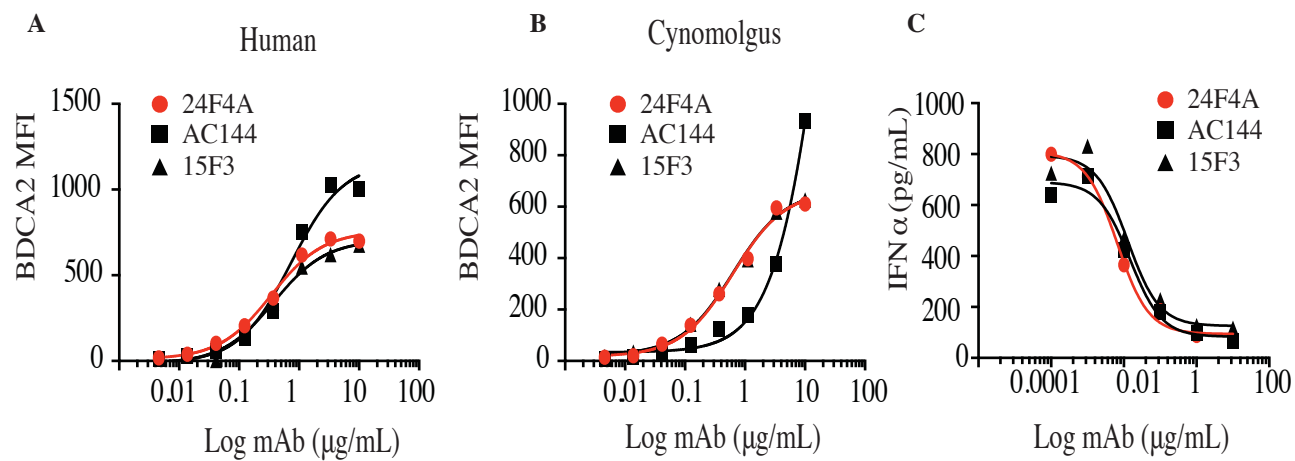

Supplement: Supplementary file 1 — Supplementary Figure S1 [file emmm0007-0464-sd1.pdf]

**Figure S2**

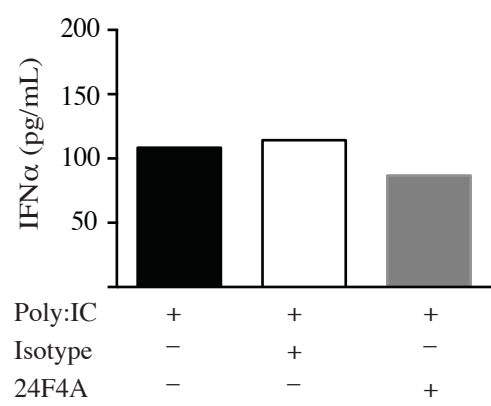

Supplement: Supplementary file 2 — Supplementary Figure S2 [file emmm0007-0464-sd2.pdf]

Figure S3

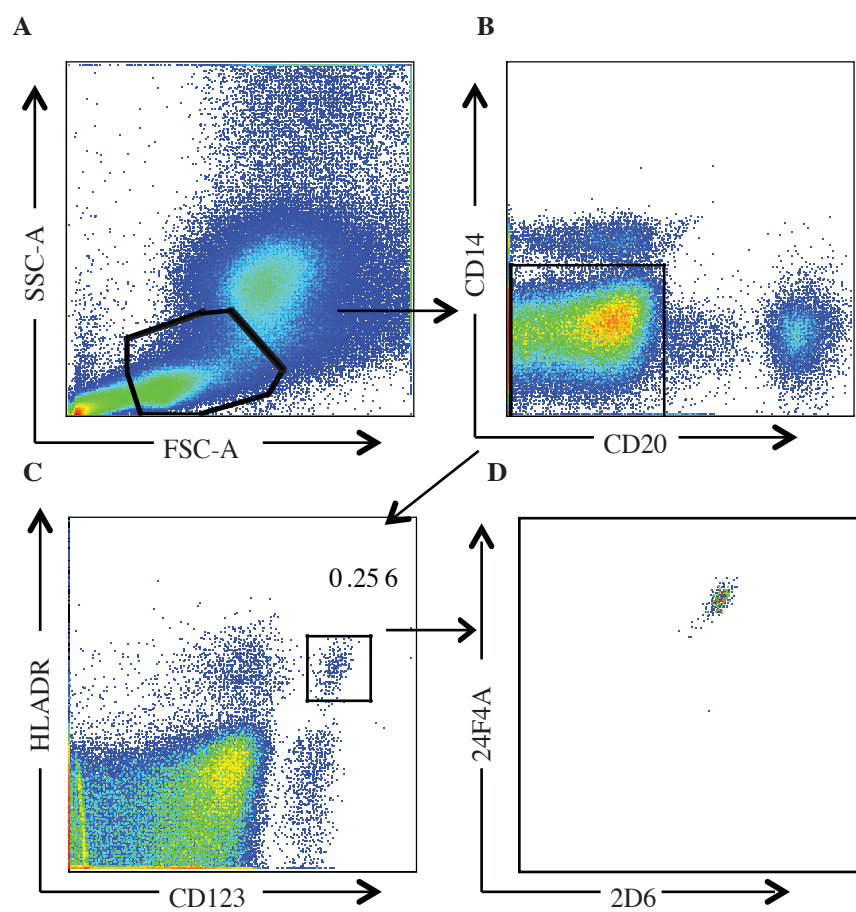

Supplement: Supplementary file 3 — Supplementary Figure S3 [file emmm0007-0464-sd3.pdf]

Figure S4

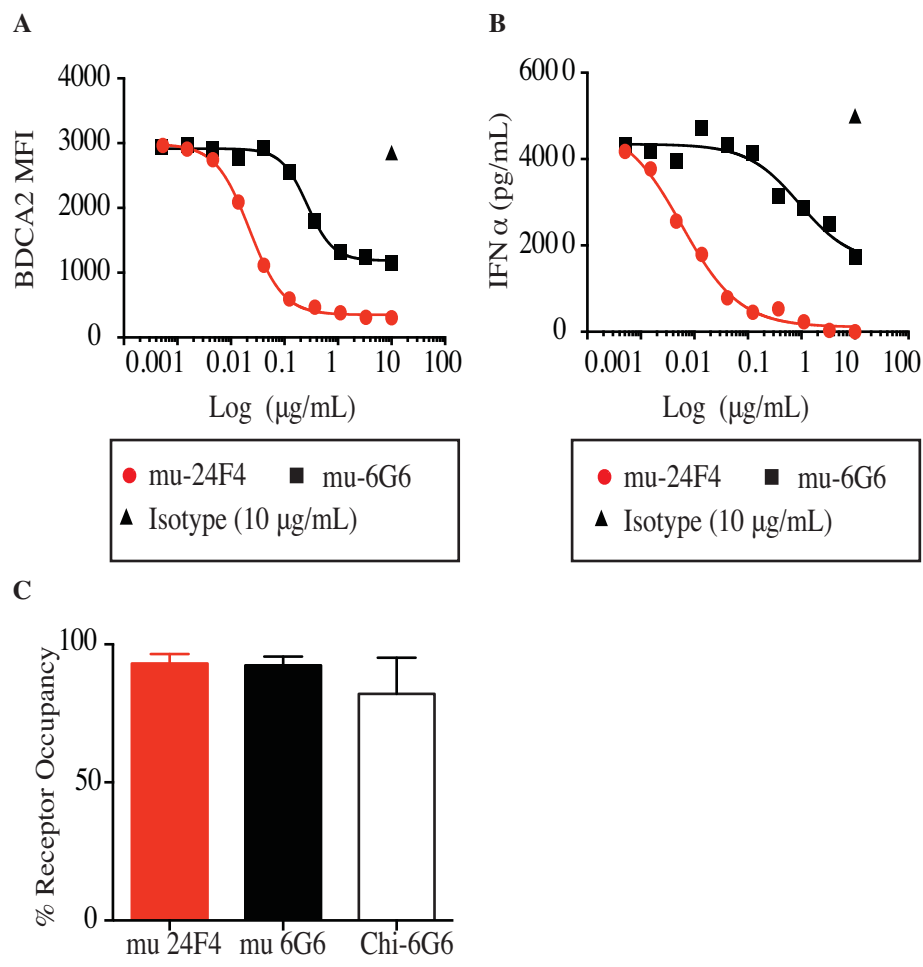

Supplement: Supplementary file 4 — Supplementary Figure S4 [file emmm0007-0464-sd4.pdf]

Figure S5

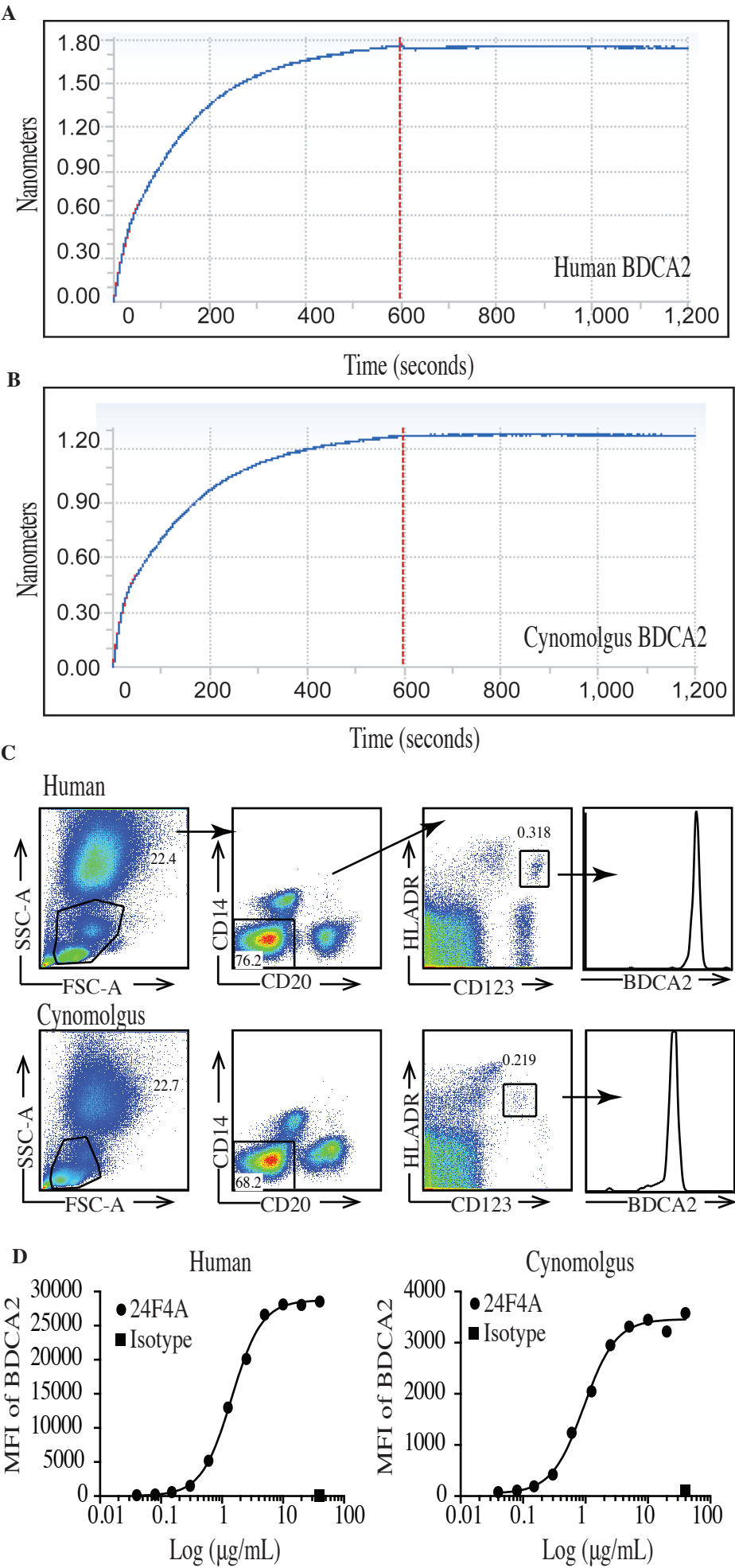

Supplement: Supplementary file 5 — Supplementary Figure S5 [file emmm0007-0464-sd5.pdf]

Figure S6

A

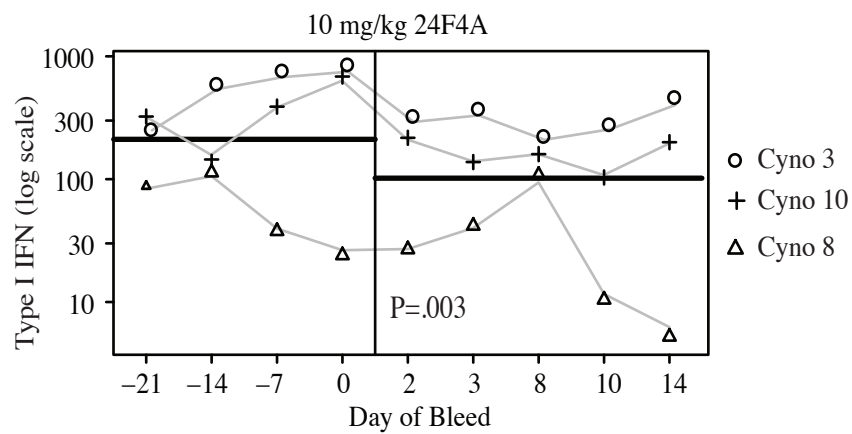

B

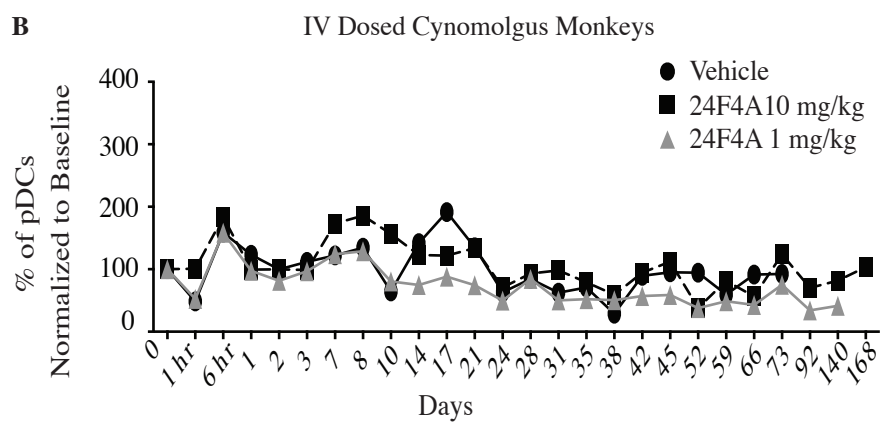

C

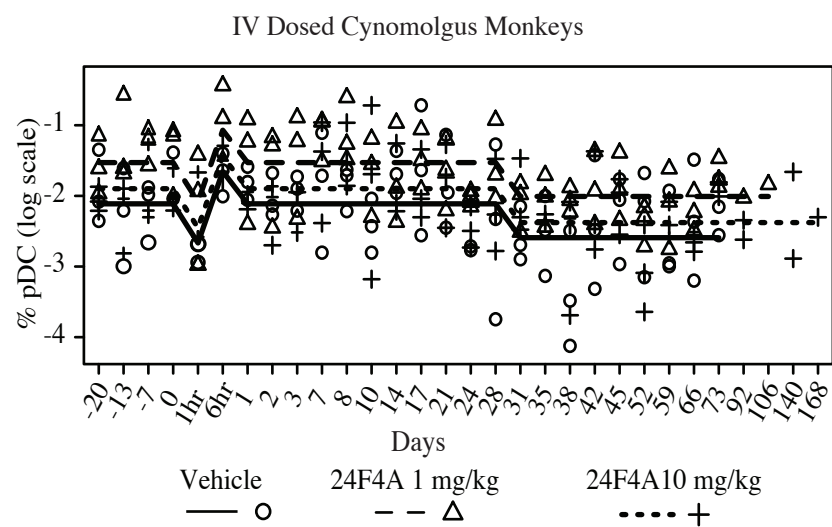

Supplement: Supplementary file 6 — Supplementary Figure S6 [file emmm0007-0464-sd6.pdf]

Figure S7

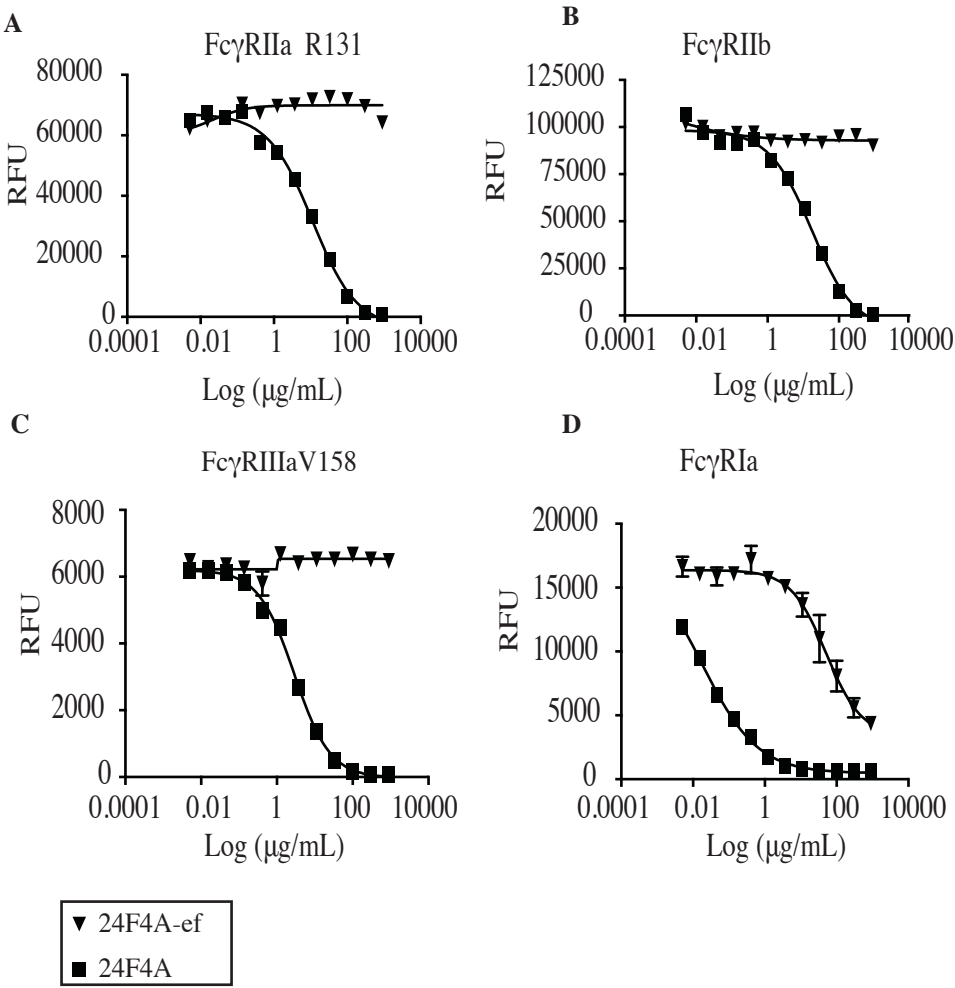

Supplement: Supplementary file 7 — Supplementary Figure S7 [file emmm0007-0464-sd7.pdf]

Figure S8

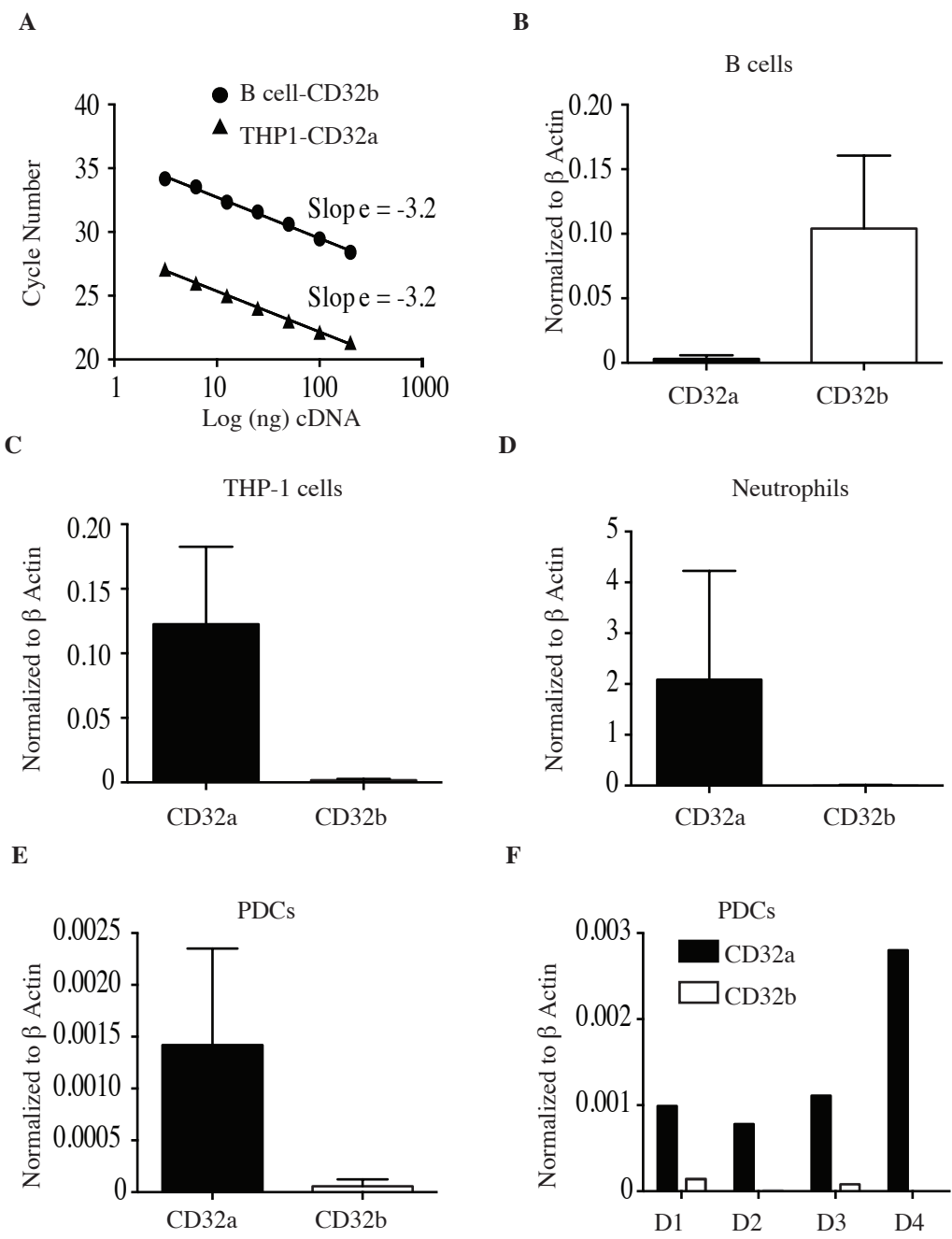

Supplement: Supplementary file 8 — Supplementary Figure S8 [file emmm0007-0464-sd8.pdf]

Figure S9

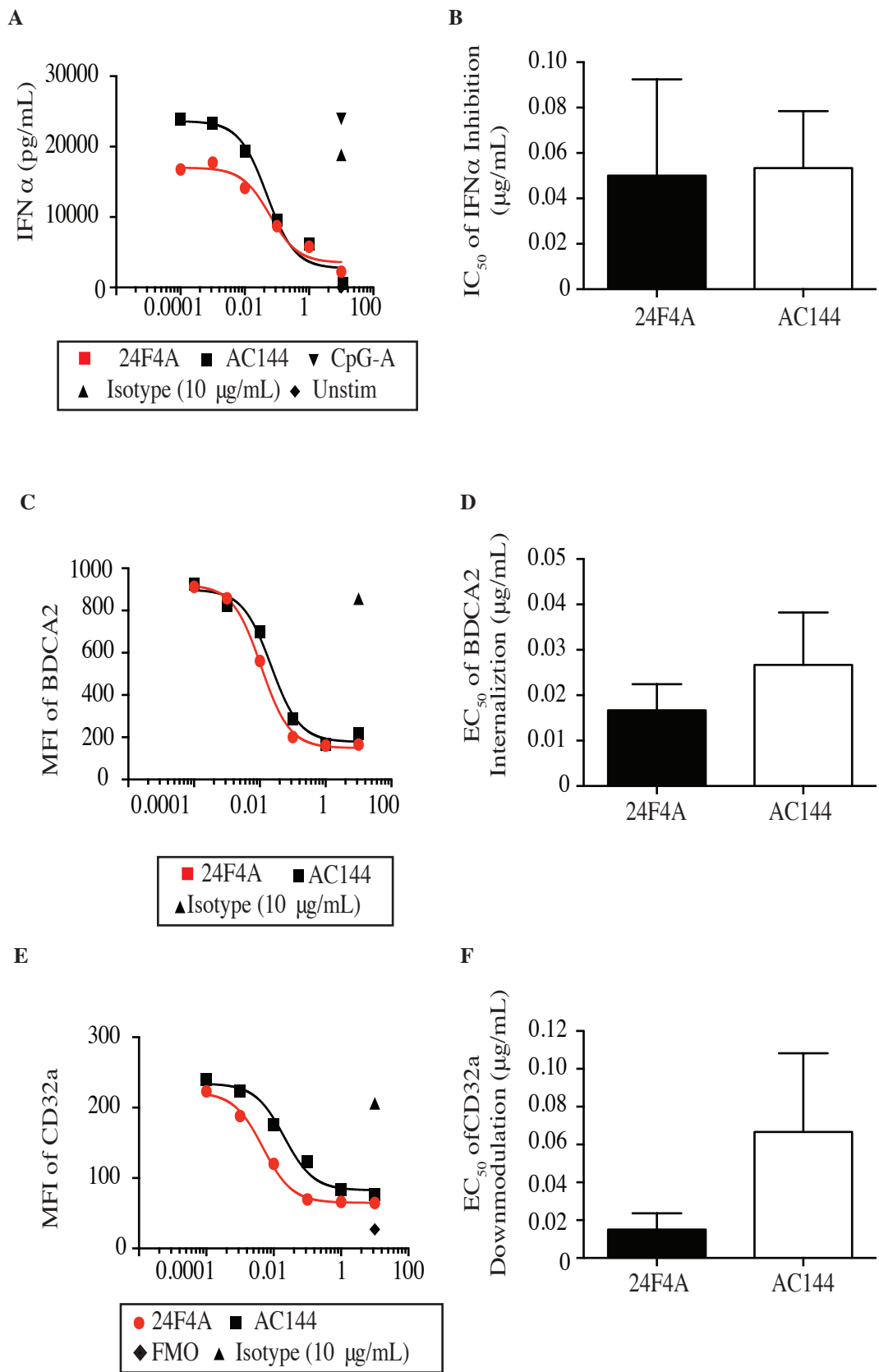

Supplement: Supplementary file 9 — Supplementary Figure S9 [file emmm0007-0464-sd9.pdf]

**Figure S10**

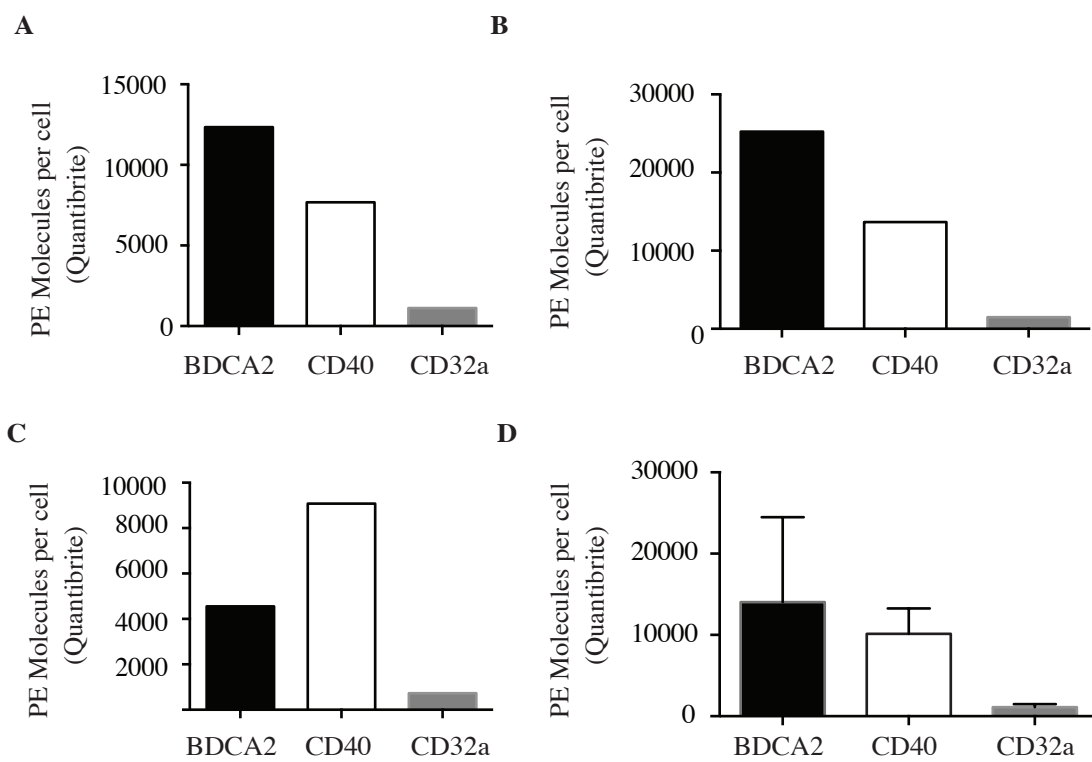

Supplement: Supplementary file 10 — Supplementary Figure S10 [file emmm0007-0464-sd10.pdf]

Figure S11

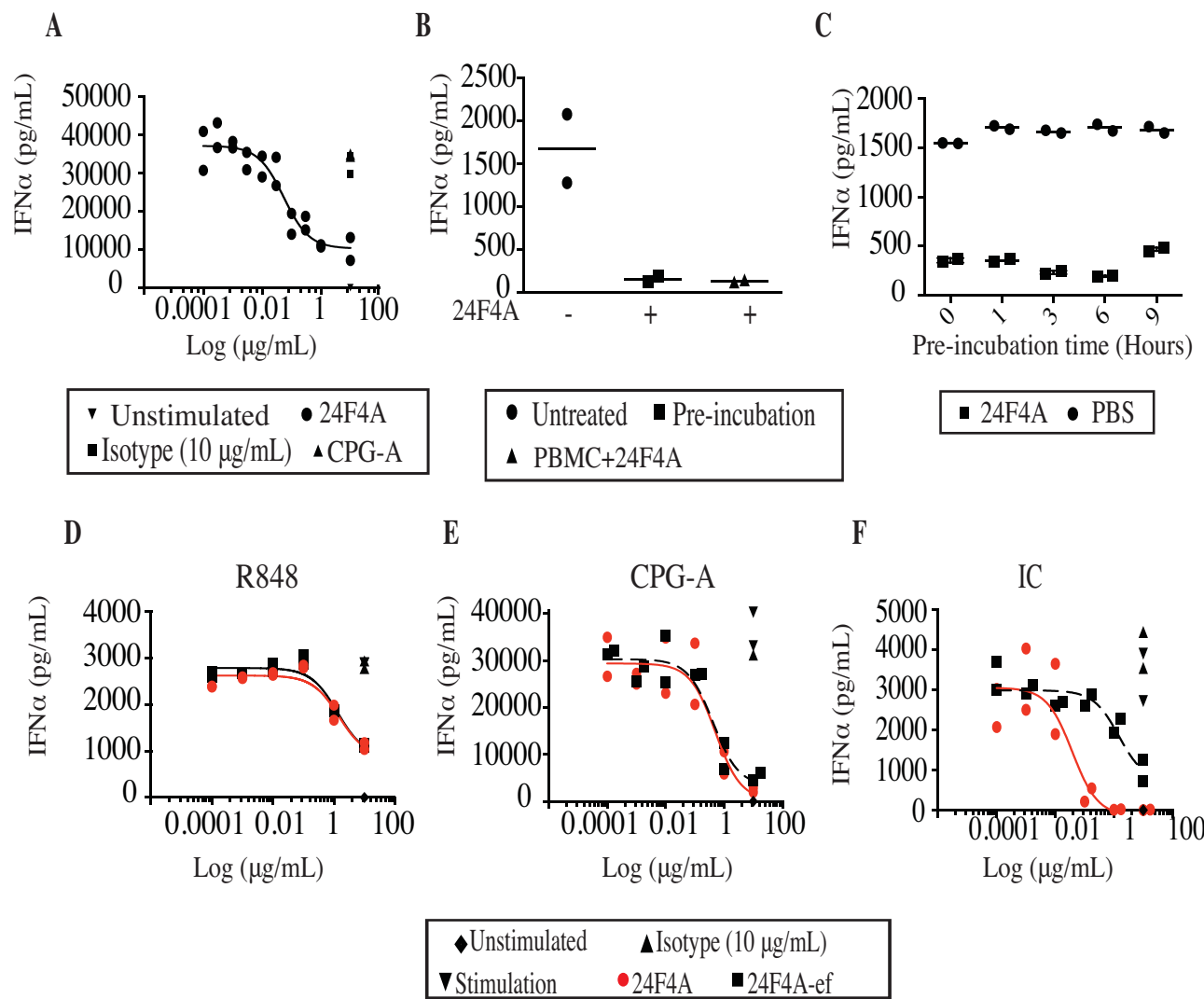

Supplement: Supplementary file 11 — Supplementary Figure S11 [file emmm0007-0464-sd11.pdf]

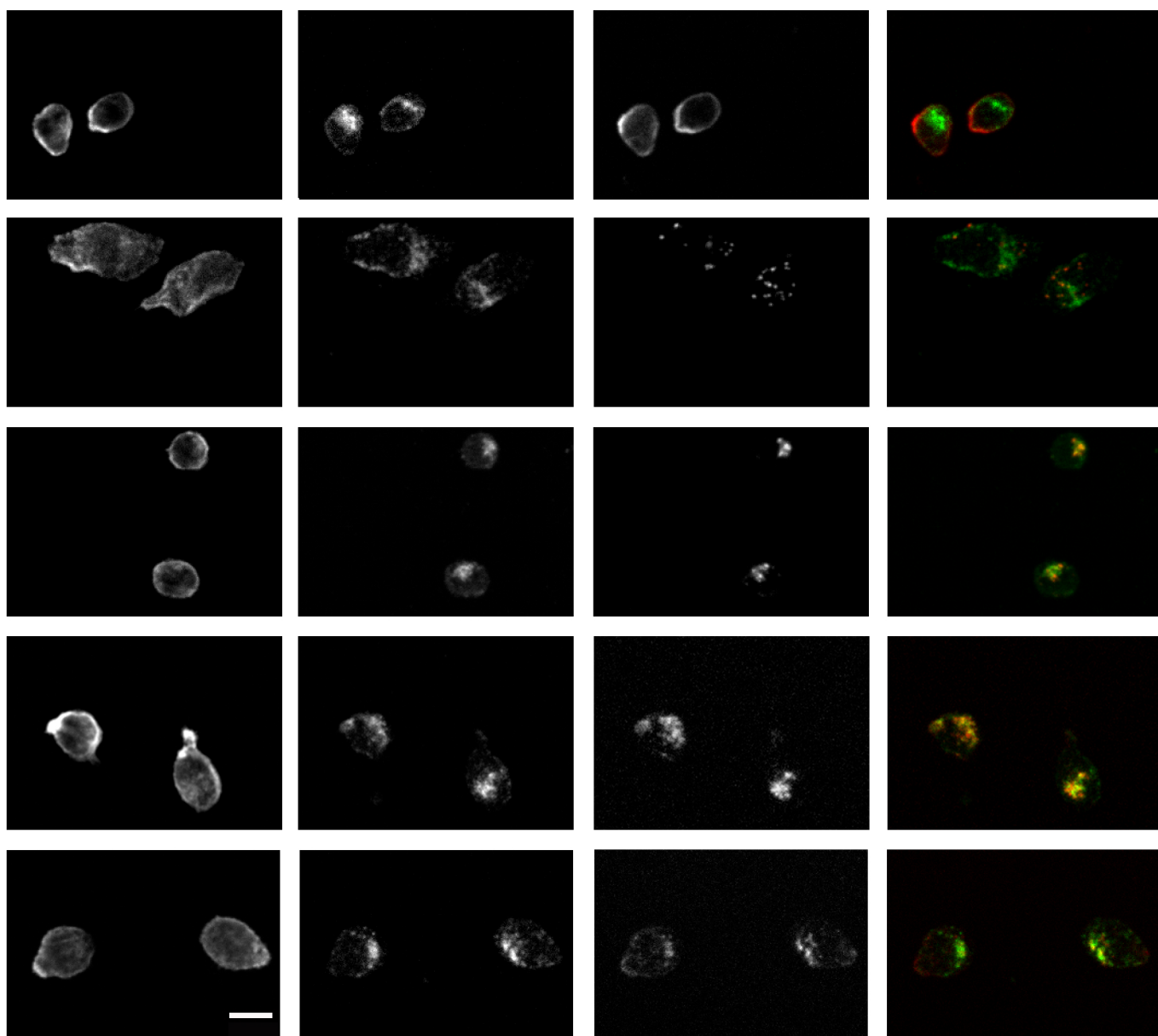

Supplement: Supplementary file 15 — Source Data for Figure 2 [file emmm0007-0464-sd15.pdf]
