# Supplementary material for: Anti-BDCA2 monoclonal antibody inhibits plasmacytoid dendritic cell activation through Fc-dependent and Fc-independent mechanisms
Source: EMBO Mol Med. 2015 Mar 11;7(4):464–76. doi: 10.15252/emmm.201404719 (PMC4403047; doi:10.15252/emmm.201404719)
Supplement: Supplementary file 12 — Supplementary Table S1 [file emmm0007-0464-sd12.pdf]

Table 1. Anti-BDCA2 antibody generation and selection

Family	mAbs	Isotype	IC50 (µg/mL) of IFNα Inhibition	EC50 of binding Human BDCA2-CHO (µg/mL)	EC50 of binding Cynomolgus BDCA2-CHO (µg/mL)	Octect affinity (nM)	Cross Block AC144	Reasons to Deprioritize
**16A8**	16A8	IgG1	0.003	0.15	0.26	7.5	yes	Binding to non transfected CHO
**24F4**	24F4	IgG1	0.008	0.34	0.65	14	yes	
	15F3	IgG1	0.01	0.36	0.71	1.5	yes	
	13H3	IgG1	0.012	0.44	0.78	6.3	yes	
**50B10**	50B10	IgG1	0.009	0.55	2.5	14	yes	Low binding to cynomolgus BDCA2
	41D12	IgG1	0.012	0.51	1.1	17	yes	
**49H9**	49H9	IgG1	0.03	1.4	1		yes	Lower potency
	47F9	IgG1	0.06	1.8	1	8	yes	
	25A11	IgG1	0.08	1.5	1.1	3.3	yes	
	31B3	IgG1	0.1	4	22	23	yes	
**Control**	6G6	IgG1	Modest Inhibition	0.22	no binding	9.1	no	
	2D6	IgG1	0.009		no binding		no	
	AC144 (myletnyi)	IgG1		0.66	11	77	yes	
