# Supplementary material for: Anti-BDCA2 monoclonal antibody inhibits plasmacytoid dendritic cell activation through Fc-dependent and Fc-independent mechanisms
Source: EMBO Mol Med. 2015 Mar 11;7(4):464–76. doi: 10.15252/emmm.201404719 (PMC4403047; doi:10.15252/emmm.201404719)
Supplement: Supplementary file 13 — Supplementary Materials and Methods, Supplementary Figure and Table Legends [file emmm0007-0464-sd13.pdf]

TABLE OF CONTENTS

SUPPLEMENTARY MATERIALS AND METHODS 2

Cloning of Human and Cynomolgus BDCA2 and Expression in CHO cells 2

Binding determination by Octet 2

MAb generation 2

MAb humanization 3

MAb expressionn and purification 4

QuantiBRITE Measurement of BDCA2, CD40 and CD32 4

QPCR Analysis of CD32a and CD32b from pDCs, THP-1 cells, Neutrophils and B cells 5

SUPPLEMENTARY TABLES 6

Table 1. Anti-BDCA2 antibody generation and selection 6

SUPPLEMENTARY FIGURES 6

Figure S1. Anti-BDCA2 mAbs bind BDCA2 and inhibit CpG-A-induced IFNα 6

Figure S2. 24F4A treatment does not affect TLR3 induced IFNα. 6

Figure S3. 24F4A and anti-BDCA2 (clone 2D6) recognize unique epitopes 6

Figure S4. 6G6 induces modest internalization of BDCA2 and modest IFNα inhibition. 6

Figure S5. 24F4A binds similarly to human and cynomolgus monkey BDCA2 7

Figure S6. 24F4A inhibits IFN-I in vivo without affecting frequency of pDCs 7

Figure S7. Relative binding affinities of 24F4A and 24F4A-G4P/G124F4A-ef to FcR 7

Figure S8. Analysis of CD32a and CD32b gene expression in human pDCs. 7

Figure S9. 24F4A or AC144 dowmmodulate of BDCA2 and CD32a on human pDCs 8

Figure S10. Comparison of BDCA2, CD40 and CD32a receptor density on pDCs. 8

Figure S11. Depiction of the IFNα levels for all replicate wells 8

SUPPLEMANTARY REFERENCES 9

SUPPLEMENTARY MATERIALS AND METHODS

Cloning of Human and Cynomolgus BDCA2 and Expression in CHO cells.

The cDNA for human FcεRIγ (NM_004106.1) and human BDCA2 (XR_242887.1) were cloned into a dual-CMV promoter vector that also carries a DHFR expression cassette. CHO DG44 cells were transfected with this plasmid using Fugene 6 and selected for incorporation in nucleoside-free, serum-free proprietary media. This stable pool was enriched by flow cytometry using commercial anti-BDCA2 antibody (Miltenyi, AC144) and cloned as single-cells. Eventually a stable clonal population demonstrating clear BDCA2 surface expression was isolated. Despite extensive cloning and selection, expression was still unstable over extended passage, and so early passage cultures were used exclusively. Due to difficulty expressing the human proteins, cynomolgus proteins were exclusively produced in transient expression. Cynomolgus FcεRIγ and BDCA2 genes were cloned from cynomolgus macaque cDNA (and were perfect matches to database sequences NM_001265840.2 and XM_005570023.1) and expressed from a similar dual-CMV promoter vector in CHO cell transient format and used 48 hours later for flow cytometry binding studies.

Binding determination by Octet

Binding of 24F4A to human and cynomolgus monkey BDCA2 ectodomain (ECD) was done by Octet Biolayer Interferometry using the FortéBio Octet Red instrument (S/N10136). Anti-human Fc Octet biosensors was equilibrated in diluent buffer (50 mM HEPES, pH 7, 100 mM NaCl, 5 mM $CaCl_2$, 1 mg/mL BSA, 0.02% Tween 20 and 0.001% azide) to establish background. 24F4A was loaded onto the anti-human Fc Octet biosensors at a concentration of 20 μg/ml. . After loading, the biosensors were once again dipped into diluent buffer to re-establish background before exposure to human or cynomolgus monkey BDCA2 ECD (2 μg/ml) for the association step. Finally the biosensors were returned to diluent buffer for the dissociation step. Data were processed using FortéBio software v.6.4.

MAb generation

92 hybridomas were generated using two approaches: gene gun immunization with a mammalian expression vector which co-expresses full-length human BDCA2 and FcεRIγ cDNAs (29 hybridomas) or immunization with soluble BDCA2-Fc (63 hybridomas). 3 mAbs were of the IgG2c isotype, 10 mAbs IgG2b isotype and the rest were of the IgG1 isotype. The mAbs were screened based on their ability to bind human and cynomolgus BDCA2 on CHO transfected cells as determined by flow cytometry (described above) and their ability to inhibit TLR9 (CpG-A)-induce IFN-I in PBMC cultures. Only 10 mAbs fulfilled these requirements and segregated into 4 primary-sequence defined families. All 10 mAbs

were determined to cross-block with AC144. Relative affinity of the antibodies was compared by Octet using an Fc BDCA2 fusion. The 24F4 mAb and a related mAb (15F3) were prioritized based on their binding capacity and high potency for inhibiting TLR9 ligand-induced IFNα by pDCs and were humanized. Fully humanized 24F4A detailed in the next section, was chosen based on its higher potency compared to 15F3 and suitable physicochemical characteristics (low levels of aggregate and solubility with high concentration).

MAb Humanization

The 24F4 antibody was humanized according to standard techniques including CDR grafting to a homologous human framework and limited back-mutations to necessary murine residues. Briefly, the canonical class of the 24F4 CDR regions was determined (Chothia et al, 1989) and the variable light and heavy chains were compared with the consensus (Kabat & Wu, 1991) and germline sequences (Matsuda et al, 1998, Brensing-Kuppers et al, 1997) for murine and human subgroups using BLAST program and internally compiled consensus and germline BLAST protein sequence databases. For the variable light chain, the closest murine germline was determined to be IGKV3-4*01 (Lefranc et al, 2009) and the closest human germline was IGKV7-3*01. For the variable heavy chain, the closest murine germline was IGHV5-6-4*01 and the closest human germline IGHV3-23*04. A structural model of the CDR-grafted humanized 24F4 was built using the PDB ID 1h0d crystal structure as the template for the frameworks. The model was assembled and refined with MOE and after analysis and comparison to public and internal antibody sequence and structure databases, two human germline frameworks, IGKV1-13*02 and IGHV3-21*01 (with consensus framework 4 regions) were chosen as the acceptor frameworks for light and heavy chains, respectively. The CDR-grafted model was examined for potential positions in which human residues might not support proper CDR conformation, and a panel of six light-chain back mutations to murine residues and sixteen heavy-chain back mutations were chosen to test experimentally. These were grouped together into three light chain variants and seven heavy chain variants and cloned into CMV-based mammalian expression vectors. Antibodies were expressed in CHO cells and conditioned supernatants screened for binding to BDCA2 antigen both in solution and on the surface of stably transfected CHO cells. The H4 and L1 variants were chosen based on their minimal number of back mutations and on the maximal affinity of the antibody they formed. An unpaired cysteine at residue 95 in CDRL3 in the 24F4 sequence was replaced in the humanized version of 24F4 by site directed mutagenesis to avoid the possibility of cysteine adducts. While serine, threonine, and alanine were each tested at this position, the alanine was chosen based on having good biophysical properties and being most "human". The humanized antibody with the alanine substitution was then referred to as 24F4A. These humanized VH and VL domains were fused to a wild-type human IgG1 (HC) and kappa (LC)

framework lacking the heavy chain C-terminal Lysine to generate wild-type 24F4A. The same VH and VL were fused to a hybrid Fc with minimal effector function bearing the CH1/hinge/CH2 from human IgG4 and the CH3 from human IgG1. In this effectorless Fc the S228 position in the hIgG4 hinge was mutated to Proline (S229P) to prevent heavy-chain scrambling and the N297 position was mutated to Q to render the Fc aglycosylated. This effector-less version of 24F4A has minimal FcR and C1q binding showing negligible C1q binding and >100-fold decrease in FcR1 binding with no apparent binding of other FcRs (Figure S5) and will be referred to in this paper as 24F4A-ef.

MAb expression and purification

Genes were inserted into CMV-based expression vectors and transfected into CHO DG44 cells and high expression clonal cell lines were isolated. For production runs, cells were grown to 5-7 million cells per ml, shifted to reduced temperature to arrest growth, and held for 10-14 days. Supernatant was clarified by centrifugation and filtration through 1.0 µm and 0.2 µm capsule filters. Purification of 24F4A and 24F4A-ef were both done by passing the cell culture supernatant over a protein A column which was depyrogenated by washing with 0.1 N NaOH followed by equilibration with PBS. After the supernatant was applied, the column was washed with PBS and 10 X PBS, and then eluted with 0.1 M sodium phosphate pH 2.8, collecting the fractions into 2 M Hepes pH 8.0 to neutralize. The peak was pooled, filtered through a 0.2 µm filter. The protein was concentrated for gel filtration. The gel filtration step was done on a Superdex 200 XK 50 column which had been depyrogenated with 0.1 N NaOH followed by equilibration with 20 mM citrate, 150 mM NaCl pH 6.0. The column was run on the AKTA Explorer, at 3.5 mL / minute collecting 12-mL fractions. The peak fractions, located by absorbance at 280 nm, were pooled, and the pool was 0.2 µm filtered. The purified antibody was quantified by UV scanning, and characterized by SDS-PAGE, analytical SEC, mass spectrometry, and binding to BDCA2-Fc fusion protein by ELISA and Octet.

QuantiBRITE Measurement of BDCA2, CD40 and CD32

Isolated pDCs were stained with saturating amounts of Phycoerythrin conjugated anti-CD32 (BD Pharmigen), anti-CD40 (BD Pharmigen) or anti-BDCA2 (Biolegend). All antibodies had a FP ratio of 1:1. QuantiBRITE PE beads (Becton Dickinson) were reconstituted with 500 µL of PBS. Cells and beads were acquired on LSR Fortessa and the MFI for each PE labeled molecule was measured. The MFIs of the low, mid/low, mid and high PE beads were also measured. PE values for CD32, CD40 and BDCA2 were calculated using a linear equation relating FL2 fluorescence to PE molecules on standardized beads per manufacturer's instructions.

QPCR Analysis of CD32a and CD32b from pDCs, THP-1 cells, Neutrophils and B cells

PDCs were isolated as described above. THP-1 cells (ATCC) were cultured with complete RMPI media. Neutrophils were isolated from human whole blood by first overlaying whole blood onto a layer of mono-poly resolving medium (MP Biomedicals) and centrifuging for 30 min at 300 g with no brake. The resulting PMN layer was collected and neutrophils were isolated following the manufacturer's protocol (Stem Cell Technologies). Total B cells were isolated according to manufacturer's protocol (miltenyi). RNA from 1-2.5x10^6 cells was extracted using an RNeasy Micro column (Qiagen) according to the manufacturer's protocol. The RNA was DNAse treated and quantified using a Nanodrop spectrophotometer. High Capacity cDNA Reverse Transcription Kit (Life Technologies) was used to generate cDNA. cDNA was diluted to 25 ng/µL in RNase-Free H_2O. 50ng of cDNA was used for each reaction. qPCR reactions were run in duplicate. Real-time quantitative PCR was performed using gene-specific fluorogenic assays containing pre-formulated primers and FAM dye-labeled probes at a 20X concentration, FCGR2A-Hs00234969_m1, FCGR2B-Hs01634996_s1, ACTB-4333762F (Applied Biosystems). 10 µL qPCR reactions of samples and gene specific assays were prepared using QuantiTect® Multiplex PCR Kit following the manufacturer's protocol.

SUPPLEMENTARY TABLE

Table 1. Anti-BDCA2 antibody generation and selection

92 hybridomas were generated using two approaches: gene gun immunization with a mammalian expression vector which co-expresses full-length human BDCA2 and FcεRIγ cDNAs (29 hybridomas) or immunization with soluble BDCA2-Fc (63 hybridomas). MAbs were screened based on their ability to bind human and cynomolgus BDCA2 on CHO transfected cells as determined by flow cytometry and their ability to inhibit TLR9 (CpG-A)-induced IFN-I in PBMC cultures. (A) 10 mAbs fulfilled these requirements and segregated into 4 primary-sequence defined families. All 10 mAbs were determined to cross-block with AC144. Relative affinity of the antibodies was compared by Octet using BDCA2-Fc fusion protein.

SUPPLEMENTARY FIGURES

Figure S1. Anti-BDCA2 mAbs bind BDCA2 and inhibit CpG-A-induced IFNα production in human PBMC. Shown is a representative plot of binding of anti-BDCA2 antibodies to human (A) and cynomolgus monkey BDCA2 expressing CHO cell lines (B). (D) Representative plot of anti-BDCA2 mediated IFNα inhibition from CpG-A stimulated PBMC.

Figure S2. 24F4A treatment does not affect TLR3 induced IFNα. PBMC from healthy donors were stimulated with 50 μg/mL of Poly:IC in the presence of 10 μg/mL of 24F4A or the isotype control. Cells were incubated for 16 hours at 37°C and 5% CO_2. ELISA was used to determine the concentration of IFNα. Bar graph represents mean IFNα from each group. Data from two independent donors are shown.

Figure S3. 24F4A and anti-BDCA2 (clone 2D6) recognize unique epitopes. Human whole blood was stained with anti-CD123, anti-CD20, anti-CD14, anti-HLADR, anti-BDCA2 (2D6) and labeled 24F4A. (A-B) PDCs were determined by first gating on lymphocytes, followed by gating on CD20 and CD14 negative cells. (C) PDCs were defined as CD123+ and HLADR+. (D) pDCs co-stained with 24F4A and αBDCA2 (2D6).

Figure S4. Anti-BDA2 mAb 6G6 induces modest internalization of BDCA2 and modest IFNα inhibition. (A-B) PBMC were treated with murine anti-BDCA2 antibodies 6G6 and 24F4. Cells were stimulated with 1μM of CpG-A and cultured for 16 hours at 37° C. (A) Flow cytometry was used to detect BDCA2 on the cell surface of pDCs and (B)ELISA was used to determine IFNα. (C) Flow cytomtery was used to determine percent receptor occupancy of murine 24F4, murine 6G6 and chimeric 6G6 after

incubation with 10 μg/mL of each antibody for 15 min at 4°C. PBMC were first treated with the unlabeled version of each mAb and then stained with the labeled version. Receptor occupancy was defined as loss of fluorescence. Error bars represent SD of mean from three independent experiments.

Figure S5. 24F4A binds similarly to human and cynomolgus monkey BDCA2. Conditioned medium containing 24F4A was assayed by Octet for binding to (A) human and (B) cynomolgus BDCA2 ectodomain. (C and D) Human or cynomolgus monkey whole blood was incubated with increasing concentrations of Alexa 647- labeled 24F4A antibody (closed circles), or Alexa 647-labeled human IgG1κ isotype control antibody (closed squares) on ice for 30 minutes. (C) pDC gating strategy ($CD14^-$ $CD20^-HLA\text{-}DR^+CD123^+$ pDCs) and mean fluorescence intensity (MFI) values of Alexafluor-647 staining of gated pDCs. (D) 24F4A binding to human pDCs from one representative experiment out of 8 independent experiments performed (right panel). 24F4A binding to cynomolgus monkey pDCs from one representative experiment out of 5 performed (left panel).

Figure S6. 24F4A mediates IFN-I inhibition *in vivo* without affecting the relative frequency of circulating pDCs. (A) Cynomolgus monkeys were dosed I.V. with 10 mg/kg 24F4A and were bled at various time points. Whole blood was stimulated with CpG-A and induction of IFN-I was measured by MXA bioassay. (B) Flow cytometry was used to determine the frequency of pDCs. The percentage of circulating pDCs post-treatment with 24F4A at10 mg/kg, 24F4A at1 mg/kg or vehicle was normalized to the pre-dose levels. At indicated time points, blood was drawn, and pDCs were identified by flow cytometry as $CD20^-CD14^-CD123^+HLA\text{-}DR^+$. (C) A linear mixed effects model for log (% pDC) values with fixed factors for dose group, time levels 1 hour, 6 hours and greater than 28 days, and with random intercept for cynomolgus monkeys. The solid line shows the fitted model.

Figure S7. Relative binding affinities of 24F4A and 24F4A-G4P/G124F4A-ef to Fc receptors. Amplified Luminescent Proximity Homogeneous Assay (ALPHA) was performed using GST kit from Perkin Elmer. IC_{50} values of 24F4A for (A), FcγR11a: 11 μg/mL, (B), FcγR11b: 17 μg/mL, (C), FcγR111a: 3 μg /mL and (D) FcγR1: 0.03 μg/mL were calculated. No binding of 24F4A G4P/G1 24F4A-ef was detected to FcγR11a, FcγR11b, and FcγR111a and binding to FcγR1 was reduced by 100-fold (A). The plates were read in an Envision plate reader (Perkin Elmer).

Figure S8. Analysis of CD32a and CD32b gene expression in human pDCs. cDNA was prepared from mRNA extracted from B cells, THP-1 cells (ATCC® TIB202) , neutrophils and pDCs. (A) The

efficiency of the probes for CD32a and CD32b was tested by serially diluting cDNA from either B cells or THP-1 cells. Shown are relative expression of CD32a and CD32b in B cells (B), THP-1 cells (C) and neutrophils (D). Error bars represent SD from three replicates. (E) Relative expression levels of CD32a and CD32b in pDCs from four individuals tested. Error bars represent SD of the mean of the four individuals tested (F) Individual plots of the relative expression of CD32a and CD32b from each of the four donors tested. The gene expression was expressed as 2Δct of βactin.

Figure S9. Treatment with 24F4A or AC144 leads to dowmmodulation of BDCA2 and CD32a on the surface of human pDCs *in vitro*. (A-F) Isolated pDCs were treated with increasing amounts of 24F4A (red circles) or AC144 (squares) and stimulated with CpG-A for 16 hours at 37°C. Flow cytometry was used to determine the levels of BDCA2 and CD32a on pDCs. ELISA was used to measure IFNα in the supernatants. (A) Plot of dose dependent anti-BDCA2- mediated IFNα inhibition from a representative donor. (B) Average IC_{50} of 24F4A or AC144- mediated IFNα inhibition (n=3 donors). (C) Representative plot of 24F4A and AC144 induced BDCA2 internalization. (D) Average EC_{50} of internalization for both 24F4A and AC144 (n=3 donors). (E) Plot of dose dependent anti-BDCA2-mediated CD32a internalization from a representative donor. (F) Average EC_{50} of internalization for both 24F4A and AC144 (n=3 donors). Triangle represents 10 μg/mL isotype control. Inverted triangle represents CpG-A alone. Diamond represents the FMO of the CD32A staining. Error bars represent SD of the mean of three independent experiments conducted.

Figure S10. Comparison of BDCA2, CD40 and CD32a receptor density on human pDCs. (A-D) Isolated pDCs were stimulated for 16 hours with CpG-A to mimic the *in vitro* conditions tested. Cells were stained separately with saturating amounts of Phycoerythrin conjugated anti- CD32, CD40 and BDCA2 antibodies (FP ratio of 1:1 for all mAbs). The number of PE molecules per cell was determined with QuantiBRITE beads. A, B and C represent the quantification for individual donors in three independent experiments. (D) Depicted is the mean and standard deviation of the three independent experiments.

Figure S11. Depiction of the IFNα levels for all replicate wells. (A-F) IFNα levels from replicate wells are shown for each experimental condition. (A) Each data point from Figure 1A shown with a curve fit line that represents the mean of the two data points. (B-C) Each data point from replicate wells from Figure 2C and D. Horizontal bars represent the mean of the two wells. (D-F) Each data point from replicate wells from Figure 4A, C and E. Curve fit is based on the mean of each data point.

SUPPLEMENTARY REFERENCES

Chothia C, Lesk AM, Tramontano A, Levitt M, Smith-Gill SJ, Air G, Sheriff S, Padlan EA, Davies D, Tulip WR, et al. (1989) Conformations of immunoglobulin hypervariable regions. *Nature* **342:** 877-883

Kabat EA, Wu TT (1991) Identical V region amino acid sequences and segments of sequences in antibodies of different specificities. Relative contributions of VH and VL genes, minigenes, and complementarity-determining regions to binding of antibody-combining sites. *Journal of immunology* **147:** 1709-1719

Lefranc MP, Giudicelli V, Ginestoux C, Jabado-Michaloud J, Folch G, Bellahcene F, Wu Y, Gemrot E, Brochet X, Lane J, Regnier L, Ehrenmann F, Lefranc G, Duroux P (2009) IMGT, the international ImMunoGeneTics information system. *Nucleic acids research* **37:** D1006-1012
